# Life after Lindane in California: Water Concentrations, Poison Control Calls Drop Following Ban

**DOI:** 10.1289/ehp.116-a128a

**Published:** 2008-03

**Authors:** Valerie J. Brown

Lindane, a persistent, highly toxic, and bioaccumulative organochlorine insecticide, was used in agriculture and as a topical treatment for human head lice and scabies beginning in the 1940s. As its toxicity became better known, manufacture and use declined in the United States; in 2002, California banned the pharmaceutical use of lindane altogether. According to a new study, that ban appears to have resulted in steep drops in concentrations of lindane in Southern California’s wastewater and a dramatic reduction in calls to the California Poison Control System **[*EHP* 116:297–302; Humphreys et al.]**.

The most common adverse effects of lindane exposure in humans include seizures, dizziness, and headaches. High levels of exposure can be fatal. Although the U.S. Environmental Protection Agency has canceled all registrations for lindane-containing compounds in agriculture, the chemical is still available by prescription as a second-line treatment for head lice in states other than California. Its continued pharmaceutical use raises concerns about its potential presence in wastewater effluent and drinking water.

The research team, part of the University of California, San Francisco, Pediatric Environmental Health Specialty Unit, examined historical lindane concentrations in several Southern California water pollution control plants and compared them before and after the ban. To assess the ban’s impact on human exposures, they analyzed lindane-related calls to California’s poison control hotline between 1998 and 2006. They searched the MediCal fee-for-service pharmacy-paid claims database and obtained national data from Verispan, a commercial health industry data tracker, to determine the number of lindane prescriptions issued. The team also conducted a random survey of pediatricians to ascertain both their awareness of the ban and their current treatment preferences for scabies and head lice.

In Los Angeles County, the average wastewater concentration of lindane in 1999 was 36 ppt. By 2006, concentrations had dropped to almost undetectable levels throughout California. In 1998, 135 per 100,000 calls to the Poison Control System concerned lindane; by 2006 such calls had declined to 2 per 100,000. Similarly, lindane prescriptions fell from 114,000 in 1997 to 34 in 2002. Medical providers reported few problems using alternative treatments such as pyrethrins.

The study authors are encouraged by their findings but note that lindane is still used in many countries, mostly in the developing world, and that every ton of lindane manufactured produces about 9 tons of toxic waste. Although the U.S. Food and Drug Administration has not banned pharmaceutical lindane in the United States, the pesticide is currently under review for inclusion in the Stockholm Convention on Persistent Organic Pollutants, which could eventually lead to a worldwide ban.

